# Quantitative [^13^N]ammonia/[^18^F]FDG PET/CT predicts left ventricular functional recovery after CABG in ischemic cardiomyopathy: An exploratory study

**DOI:** 10.1371/journal.pone.0358657

**Published:** 2026-09-21

**Authors:** Fukai Zhao, Zekun Pang, Haoran Guo, Yue Chen, Jiao Wang, Bailing Hsu, Jianming Li

**Affiliations:** 1 Department of Nuclear Medicine, TEDA International Cardiovascular Hospital, Clinical College of Cardiovascular Diseases, Tianjin Medical University, Tianjin, China; 2 Nuclear Science and Engineering Institute, University of Missouri-Columbia, Columbia, Missouri, United States of America; Fondazione Policlinico Universitario Agostino Gemelli IRCCS, ITALY

## Abstract

**Background:**

In patients with ischemic cardiomyopathy (ICM), accurate preoperative identification of viable myocardium is clinically important to identify those who are likely to benefit from coronary artery bypass grafting (CABG). Although the PET perfusion–metabolism mismatch index has long been used to assess myocardial viability, its value in predicting contractile recovery remains controversial. Quantitative metabolic indices based on standardized uptake value (SUV) may provide complementary prognostic information.

**Methods:**

This single-center retrospective cohort study included 53 patients with ICM who underwent preoperative [^13^N]ammonia/[^18^F]FDG PET/CT followed by isolated CABG. Conventional perfusion–metabolism indices were compared with SUV-based indices. The evaluated outcomes were postoperative increases in left ventricular ejection fraction (ΔLVEF) of ≥5% and ≥10% on follow-up echocardiography, and time to first major adverse cardiovascular event (MACE). Each PET parameter was evaluated in a separate multivariable Firth-penalized logistic regression model adjusted a priori for age, baseline LVEF, history of coronary artery disease, and diabetes. The predictive performance was assessed by receiver operating characteristic analysis, and time to MACE by Cox regression with the adjustment of multiple testing and model optimism.

**Results:**

Of the 53 patients, 32 (60.4%) achieved ΔLVEF ≥5% and 25 (47.2%) achieved ΔLVEF ≥10%. The infarct-to-non-infarct SUV ratio (SUVR) was associated with ΔLVEF ≥5% (odds ratio [OR] 1.94 per 0.1-unit increase, 95% confidence interval [CI] 1.28–3.25; *P* < 0.001; *q* = 0.003) and ΔLVEF ≥10% (OR 1.53, 95% CI 1.11–2.29; *P* = 0.004; *q* = 0.035). For the prediction of ΔLVEF ≥5%, SUVR had a higher area under the curve (AUC) than the mismatch index (0.83 vs 0.62; DeLong *P* = 0.0049; *q* = 0.0147). For the prediction of ΔLVEF ≥10%, the AUCs did not differ (0.73 and 0.63; DeLong *P* = 0.200; *q* = 0.383). No individual PET parameter was associated with time to first MACE in adjusted Cox models.

**Conclusions:**

SUVR showed greater discrimination for postoperative ΔLVEF ≥5% than the conventional perfusion–metabolism mismatch index. This finding supports SUVR as a quantitative adjunct to conventional PET viability assessment and warrants further multicenter validation.

## Introduction

Ischemic cardiomyopathy (ICM), an advanced manifestation of coronary artery disease (CAD), is a leading cause of heart failure. It is characterized predominantly by left ventricular systolic dysfunction due to chronic myocardial hypoperfusion. This condition can lead to reduced cardiac output, impaired perfusion of organs and peripheral tissues, and systemic venous congestion, ultimately contributing to multiorgan dysfunction, poor quality of life, and adverse prognosis [1]. Impaired cardiac contractility in ICM is attributable not only to necrotic scar tissue but also to “hibernating myocardium,” defined as viable myocardium with chronically reduced or absent contractility because of persistent hypoperfusion. Hibernating myocardium is functionally recoverable and therefore represents a therapeutic target for improving contractility and clinical outcomes [2]. Coronary artery bypass grafting (CABG), a surgical revascularization strategy for advanced CAD, can restore blood flow to hibernating myocardial territories and may promote favorable ventricular remodeling, regional contractility, and left ventricular ejection fraction (LVEF) recovery [3,4]. Improved cardiac function after CABG may enhance quality of life and reduce adverse cardiovascular events. Contractile recovery after CABG depends on several factors, including the extent of viable myocardium, baseline LVEF, age, frailty, and comorbidity burden [5,6]. Preoperative identification of patients most likely to benefit from CABG is therefore clinically important.

Positron emission tomography (PET) using resting [^13^N]ammonia myocardial perfusion imaging and [^18^F]FDG metabolic imaging is a widely recognized noninvasive approach for assessing myocardial viability [7]. By characterizing myocardial perfusion–metabolism patterns, PET can identify potentially recoverable myocardium, manifested as reduced perfusion with preserved or increased glucose metabolism, the so-called “perfusion–metabolism mismatch pattern” [8]. Although the perfusion–metabolism mismatch index has been widely applied in clinical practice for decades to predict functional recovery, its predictive value remains debated. Several large clinical trials, including PARR-2 and STICH, have reported negative or inconclusive results, challenging the role of viability assessment in guiding revascularization and improving clinical outcomes [9,10]. The inconclusive findings may partly reflect limitations of conventional relative assessments of myocardial metabolism. SUV-based quantification, initially developed in other nuclear imaging applications, may provide a more objective assessment of myocardial radiotracer distribution and complement conventional viability assessment [11–13].

To our knowledge, SUV-based metabolic indices have not been evaluated for postoperative functional recovery after CABG in patients with ICM. We therefore compared SUV-based indices with conventional PET viability indices for their associations with LVEF recovery and explored their relationships with short-term adverse cardiovascular events.

## Methods

### Study subjects

This single-center retrospective study screened consecutive patients who underwent [^13^N]ammonia/[^18^F]FDG PET/CT myocardial viability assessment at the Department of Nuclear Medicine, TEDA International Cardiovascular Hospital, between April 2016 and March 2022. Patients were eligible for inclusion if they met the following criteria: (1) documented ICM with a baseline left ventricular ejection fraction (LVEF) ≤ 40% on transthoracic echocardiography performed within 30 days before the PET scan; (2) underwent isolated coronary artery bypass grafting (CABG) within 30 days after PET imaging; and (3) had an available postoperative echocardiogram, clinically targeted for approximately 6–12 months after CABG, together with corresponding clinical follow-up data for assessment of short-term major adverse cardiovascular events (MACE); the actual CABG-to-echocardiography interval was recorded for each included patient. Patients were excluded if they had a non-ischemic etiology, recent acute coronary syndrome or myocardial infarction within 4 weeks before PET, prior or repeat CABG, or inadequate PET/CT image quality. Prior percutaneous coronary intervention (PCI) was not an exclusion criterion. Written informed consent specifically for the PET/CT imaging procedure and standard clinical data collection was obtained from all patients prior to the scan. Clinical, imaging, echocardiographic, and follow-up data were accessed for research purposes on August 19, 2024. The retrospective analysis of the clinical and imaging data was approved by the Ethics Committee of TEDA International Cardiovascular Hospital, with a waiver for additional study-specific informed consent (Approval number: 2024-0722-2). This study was conducted in accordance with the Declaration of Helsinki.

### PET/CT myocardial viability imaging

All 53 patients underwent preoperative [^13^N]ammonia perfusion and [^18^F]FDG metabolic PET/CT for myocardial viability assessment. Both radiotracers were produced by the in-house medical cyclotron (MINItrace Qilin, GE HealthCare, Chicago, IL, USA) and synthesized using the TraceLab automated synthesis system, with radiochemical purity ≥ 95%. Acquisitions were performed on a Discovery Elite PET/CT system (NM690, GE HealthCare, Milwaukee, WI, USA) equipped with time-of-flight capability. A low-dose CT scan (120 kV, 20 mA) was used for attenuation correction. Resting perfusion and metabolic imaging were performed consecutively on the same day. Images were reconstructed using an ordered-subset expectation maximization algorithm and corrected for attenuation, scatter, random coincidences, and geometric efficiency.

Before [¹⁸F]FDG PET imaging, patients fasted for at least 6 hours. Baseline blood glucose was measured, and preparation followed a local oral glucose–insulin protocol stratified by diabetes status. Oral glucose (5–40 g) was administered according to the baseline value, and blood glucose was remeasured approximately 60 minutes later. Intravenous insulin (0.5–4 IU) was given when indicated, with repeat glucose testing after 30–40 minutes. [¹⁸F]FDG (111–185 MBq) was injected when the protocol-defined criterion was met, generally at 7–8 mmol/L or with a clear downward trend; at 8–9 mmol/L, tracer injection was permitted after low-dose insulin. A small amount of food was permitted 15–20 minutes after tracer injection. ECG-gated PET began 60 minutes after injection and used the same CT and PET settings as the perfusion scan, with 8 frames per cardiac cycle and an 8-minute acquisition.

### Image processing and analysis

Reconstructed [^13^N]ammonia and [^18^F]FDG PET images, including attenuation and other physical corrections, were imported into MyoFlowQ (Beijing Lark Cloud Biomedicals, Beijing, China) and reoriented to standard short-axis slices. [^18^F]FDG images were converted to SUV using image-derived activity concentration (Bq/mL), injected activity (Bq), and body weight. A valve-plane cut line and an ellipsoidal left-ventricular sampling surface were used to generate aligned perfusion and metabolic polar maps. Pixels with <50% of the maximum [^13^N]ammonia polar-map intensity formed the prespecified low-perfusion (‘infarct’) region; the remaining pixels formed the non-infarct region. This operational relative-uptake threshold was supported by prior [^13^N]ammonia work [14]. The masks were transferred to the [^18^F]FDG map to measure SUV_infarct_ and SUV_non-infarct_. A 10 × 10 × 20 mm^3^ region in the left atrial cavity provided the blood-pool reference (SUV_blood-pool_). SUVR was calculated as SUV_infarct_/SUV_non-infarct_; NSUV_infarct_ and NSUV_non-infarct_ were calculated relative to SUV_blood-pool_.

**Fig 1:**
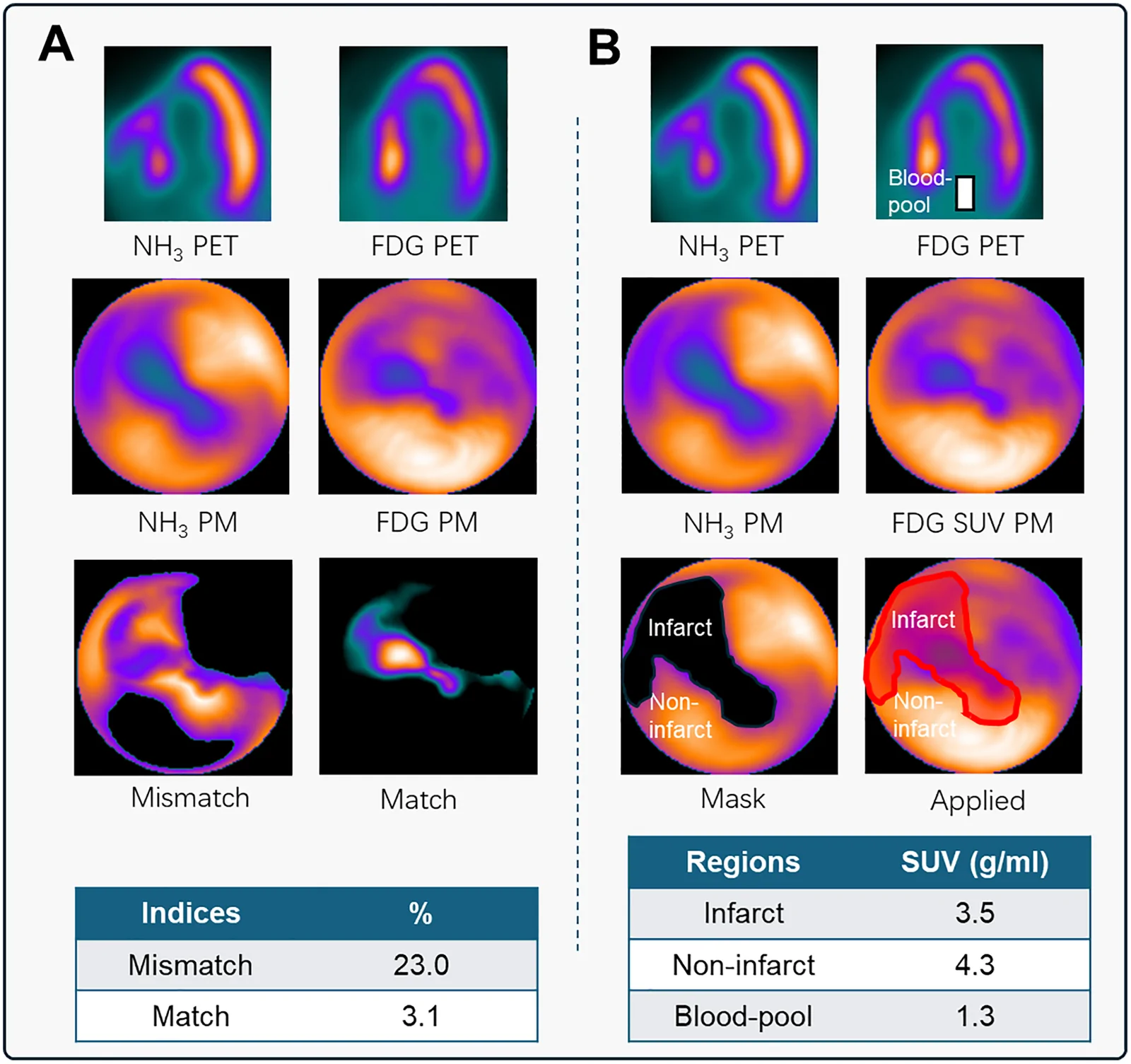
PET image-processing workflow for conventional and SUV-based measurements. (A) Mismatch and match indices used to calculate MMR. (B) Mean SUV measurements in low-perfusion, non-infarct, and left atrial blood-pool regions; the red outline denotes the low-perfusion region transferred from [^13^N]ammonia perfusion to [^18^F]FDG metabolic images. *Abbreviations: MMR, mismatch-to-match ratio; PET, positron emission tomography; PM, polar map; SUV, standardized uptake value.*

Conventional mismatch and match indices, each expressed as a percentage of LV myocardium, were obtained using the PARR-1 method [15], and the mismatch-to-match ratio (MMR) was calculated. The main workflow is shown in Fig 1, with detailed segmentation and ROI/formula diagrams in S1 Fig and S2 Fig. Two experienced nuclear medicine physicians jointly reviewed image registration, myocardial contours, ROI placement, and final measurements while blinded to clinical history, surgical details, and postoperative outcomes; discrepancies were resolved by consensus. MyoFlowQ has not undergone published head-to-head validation against QGS/QPS for this workflow.

### Follow-up and outcomes

ΔLVEF was defined as follow-up LVEF minus baseline LVEF. The evaluated outcomes were an absolute ΔLVEF ≥5%, an absolute ΔLVEF ≥10% [16], and time to first MACE after CABG. MACE was defined as rehospitalization for cardiovascular causes, worsening heart failure, recurrent angina, myocardial infarction, arrhythmia, or cardiac death. Follow-up time was calculated from CABG to the first documented MACE or, for patients without MACE, to the last available clinical follow-up.

Baseline LVEF was obtained from the echocardiogram closest to the PET scan. Follow-up echocardiography was clinically targeted for approximately 6–12 months after CABG, and the actual interval was recorded in days. Standard two-dimensional transthoracic echocardiographic images were acquired from conventional views, including the apical four-chamber and two-chamber views. LVEF was calculated using the modified biplane Simpson method, according to the recommendations of the American Society of Echocardiography and the European Association of Cardiovascular Imaging [17].

### Statistical analysis

Continuous variables are presented as mean ± standard deviation (SD) or median [interquartile range (IQR)], and categorical variables as counts and percentages. Group comparisons were performed using the independent-samples t test, Wilcoxon rank-sum test, or Fisher exact test, as appropriate. To evaluate associations with postoperative LVEF recovery while reducing small-sample bias and avoiding multicollinearity, separate Firth-penalized logistic regression models were constructed for each PET-derived parameter. Each model was adjusted a priori for age, baseline LVEF, history of CAD (prior myocardial infarction and/or prior PCI), and diabetes. Sensitivity analyses additionally adjusted for the CABG-to-follow-up echocardiography interval and, among patients with complete angiographic data, three-vessel CAD. Receiver operating characteristic (ROC) curves were used to assess discrimination and were compared using DeLong tests. Exploratory cutoffs and corresponding sensitivities and specificities were identified using the Youden index; the adjusted SUVR models and cutoffs were internally validated with 1,000 bootstrap resamples. For time to first MACE, PET parameters were evaluated in separate Cox proportional hazards models adjusted for age and baseline LVEF, with proportional hazards assessed using Schoenfeld residuals. Kaplan–Meier curves were generated for descriptive visualization after dichotomization at the sample median. Benjamini–Hochberg false-discovery-rate correction was applied separately within each outcome and analysis family. All tests were two-sided, with *P* < 0.05 and *q* < 0.05 considered statistically significant. Analyses were performed using R version 4.5.1 and GraphPad Prism version 10.6.0.

## Results

### Baseline patient characteristics and changes in LVEF

**Fig 2:**
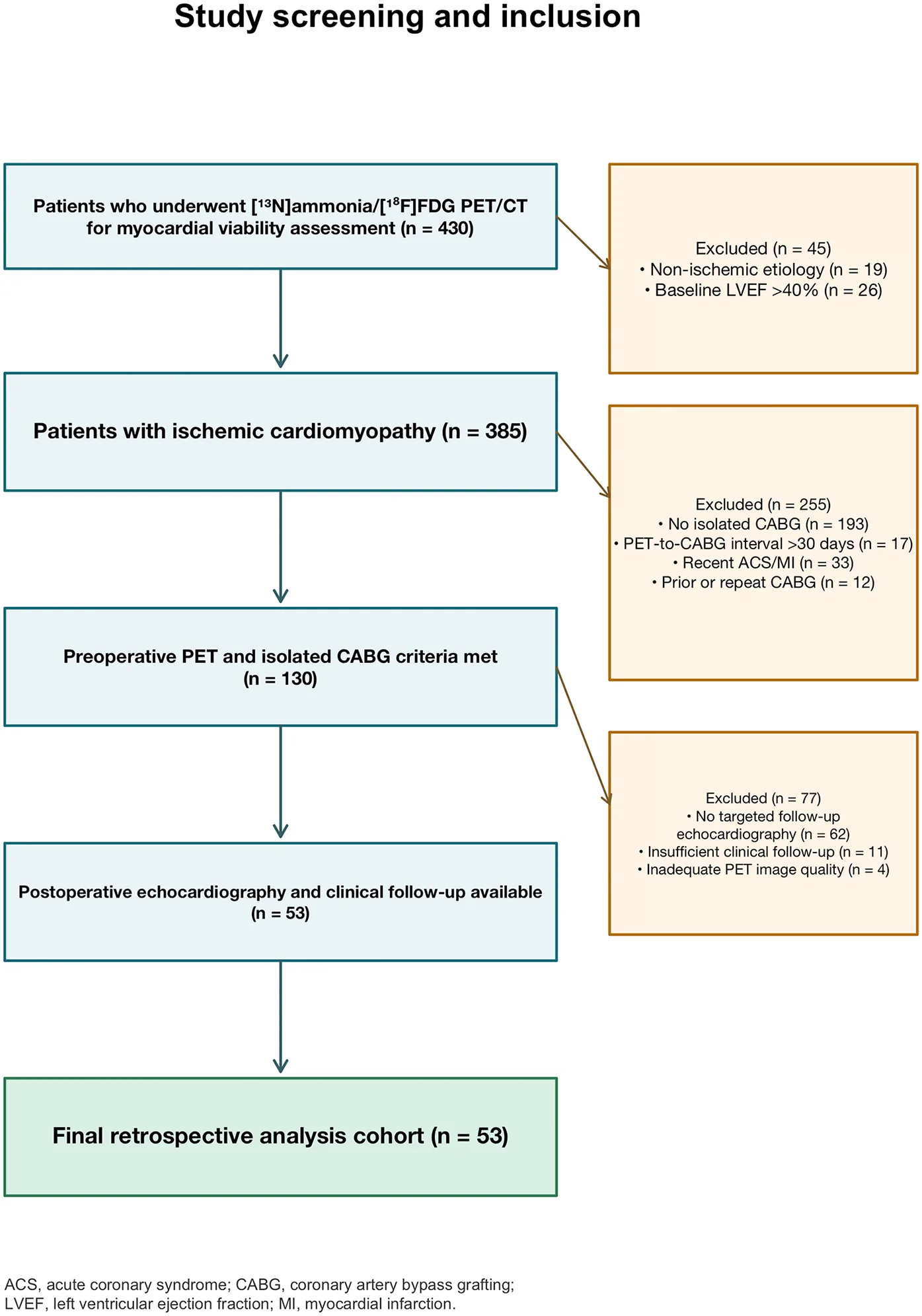
Flow diagram of patient screening, exclusions, and final cohort inclusion. **Of 430 patients screened, 53 were included in the analysis.** Abbreviations: ACS, acute coronary syndrome; CABG, coronary artery bypass grafting; ICM, ischemic cardiomyopathy; LVEF, left ventricular ejection fraction; MACE, major adverse cardiovascular event; MI, myocardial infarction; PET/CT, positron emission tomography/computed tomography.

From April 2016 to March 2022, 430 consecutive patients referred for [^13^N]ammonia/[^18^F]FDG PET/CT myocardial viability assessment were screened. After the eligibility criteria were applied (Fig 2), 45 were excluded because of non-ischemic disease or baseline LVEF >40%, 255 because they did not meet the isolated-CABG, timing, recent-event, or prior-CABG criteria, and 77 because follow-up echocardiography, clinical follow-up, or adequate PET images were unavailable. The final cohort comprised 53 patients with ICM (mean age 57.60 ± 11.30 years; 50 [94.3%] men). The mean preoperative LVEF was 34% ± 4%, and 32 patients (60.4%) achieved ΔLVEF ≥5%. Baseline characteristics are shown in Table 1.

**Table 1:** Baseline characteristics and PET parameters according to postoperative ΔLVEF ≥5% after CABG.

Variables	All patients (*n* = 53)	ΔLVEF <5% (*n* = 21)	ΔLVEF ≥5% (*n* = 32)	*P* value
Demographic characteristics				
Age (years)	57.60 ± 11.30	58.71 ± 10.94	56.88 ± 11.64	0.59
Sex, male [*n* (%)]	50 (94.3)	20 (95.2)	30 (93.8)	1.00
BMI, kg/m²	26.48 ± 3.94	26.78 ± 3.67	26.29 ± 4.16	0.88
Hypertension, *n* (%)	25 (47.2)	13 (61.9)	12 (37.5)	0.10
Diabetes mellitus, *n* (%)	18 (34.0)	5 (23.8)	13 (40.6)	0.25
Dyslipidemia, *n* (%)	2 (3.8)	0 (0.0)	2 (6.2)	0.51
Smoking history, *n* (%)	34 (64.2)	15 (71.4)	19 (59.4)	0.40
Family history, *n* (%)	5 (9.4)	2 (9.5)	3 (9.4)	1.00
Alcohol history, *n* (%)	24 (45.3)	10 (47.6)	14 (43.8)	1.00
History of CAD				
Pre-CABG MI, *n* (%)	29 (54.7)	15 (71.4)	14 (43.8)	0.06
Previous PCI, *n* (%)	13 (24.5)	8 (38.1)	5 (15.6)	0.10
Coronary angiography, *n* (%)¹	43 (81.1)	18 (85.7)	25 (78.1)	0.72
1-vessel CAD	6 (14.0)	4 (22.2)	2 (8.0)	0.22
2-vessel CAD	5 (11.6)	3 (16.7)	2 (8.0)	0.63
3-vessel CAD	32 (74.4)	11 (61.1)	21 (84.0)	0.16
Baseline LVEF, %	33.91 ± 4.35	33.84 ± 4.50	34.22 ± 3.73	0.93
CABG-to-follow-up echo interval, days	317 [248–353]	333 [259–345]	310 [228–365]	0.85
PET parameters				
Mismatch index, %	3 [1 8]	2 [1 6]	4 [2 10]	0.14
Match index, %	6 [2 11]	11 [5 22]	4 [2 7]	**0.01**
MMR	0.50 [0.20, 1.73]	0.31 [0.06, 0.50]	0.77 [0.35, 2.90]	**<0.01**
SUV_infarct_	4.70 [3.90, 6.50]	4.30 [3.30, 5.00]	5.25 [4.07, 6.90]	**0.03**
SUV_non-infarct_	6.70 [5.00, 8.80]	7.00 [5.50, 8.70]	6.65 [4.20, 8.93]	0.88
SUV_blood-pool_	1.60 [1.30, 2.00]	1.80 [1.30, 2.30]	1.55 [1.30, 1.83]	0.30
NSUV_infarct_	3.15 [2.28, 4.00]	2.33 [1.92, 3.14]	3.56 [2.84, 4.53]	**<0.01**
NSUV_non-infarct_	4.41 [2.83, 5.57]	4.32 [2.78, 5.67]	4.69 [3.31, 5.52]	0.65
SUVR	0.75 [0.60, 0.89]	0.60 [0.57, 0.68]	0.85 [0.71, 0.95]	**<0.01**

Note. Continuous variables are presented as mean ± SD or median [IQR], and categorical variables as *n* (%).

*P* values were obtained using the independent-samples t test, Wilcoxon rank-sum test, or Fisher exact test, as appropriate; bold values indicate *P* < 0.05.

¹ Coronary angiography data were available for 43 patients (ΔLVEF <5%, *n* = 18; ΔLVEF ≥5%, *n* = 25).

*Abbreviations: BMI, body mass index; CABG, coronary artery bypass grafting; PCI, percutaneous coronary intervention; CAD, coronary artery disease; IQR, interquartile range; LVEF, left ventricular ejection fraction; MI, myocardial infarction; MMR, mismatch-to-match ratio; NSUV, normalized standardized uptake value; PET, positron emission tomography; SD, standard deviation; SUV, standardized uptake value; SUVR, infarct-to-non-infarct SUV ratio.*

**Fig 3:**
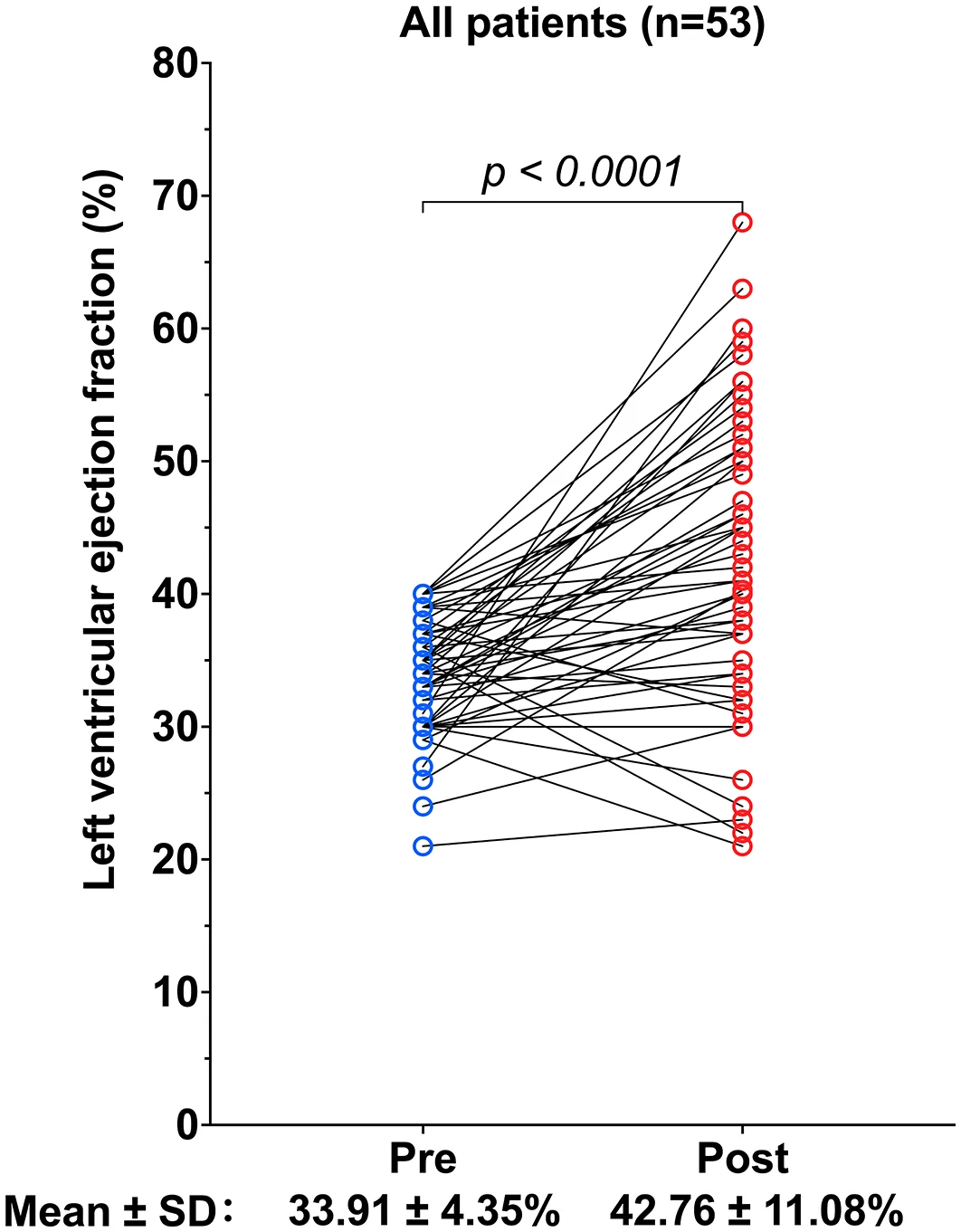
Individual changes in LVEF from baseline to follow-up after CABG (*n* = 53). Each line connects paired measurements from one patient; the *P* value is from the paired comparison. Abbreviations: CABG, coronary artery bypass grafting; LVEF, left ventricular ejection fraction; SD, standard deviation.

Overall LVEF increased from 33.91 ± 4.35% to 42.76 ± 11.08% (paired *P* < 0.001) (Fig 3). The CABG-to-follow-up echocardiography interval was 317 days [IQR 248–353; range 181–394]. It did not differ by either LVEF outcome (*P* = 0.85 and *P* = 0.87) and was not correlated with continuous ΔLVEF (Spearman *ρ* = −0.003; *P* = 0.982). Thirty-two patients (60.4%) achieved ΔLVEF ≥5%, and 25 (47.2%) achieved ΔLVEF ≥10%.

### Relationship Between PET Quantitative Parameters and Cardiac Function Recovery

Compared with patients with ΔLVEF <5%, those with ΔLVEF ≥5% had lower match-index values, higher MMR, higher SUV_infarct_, higher NSUV_infarct_, and higher SUVR (Table 1). A similar pattern was observed for ΔLVEF ≥10% (Table 2). After Benjamini–Hochberg correction across the nine PET comparisons, the match index, MMR, NSUV_infarct_, and SUVR remained significant for ΔLVEF ≥5%; MMR and SUVR remained significant for ΔLVEF ≥10% (S1 Table).

**Table 2:** Baseline characteristics and PET parameters according to postoperative ΔLVEF ≥10% after CABG.

Variables	All patients (*n* = 53)	ΔLVEF <10% (*n* = 28)	ΔLVEF ≥10% (*n* = 25)	*P value*
*Demographic characteristics*				
Age (years)	61 [50, 66]	59 [51, 66]	63 [45, 67]	0.80
Sex, male [*n* (%)]	50 (94.3)	26 (92.9)	24 (96.0)	1.00
BMI, kg/m²	26.48 ± 3.94	26.73 ± 3.78	26.21 ± 4.18	0.63
Hypertension, *n* (%)	25 (47.2)	15 (53.6)	10 (40.0)	0.41
Diabetes mellitus, *n* (%)	18 (34.0)	8 (28.6)	10 (40.0)	0.40
Dyslipidemia, *n* (%)	2 (3.8)	0 (0.0)	2 (8.0)	0.22
Smoking history, *n* (%)	34 (64.2)	19 (67.9)	15 (60.0)	0.58
Family history, *n* (%)	5 (9.4)	3 (10.7)	2 (8.0)	1.00
Alcohol history, *n* (%)	24 (45.3)	14 (50.0)	10 (40.0)	0.58
*History of CAD*				
Pre-CABG MI, *n* (%)	29 (54.7)	17 (60.7)	12 (48.0)	0.41
Previous PCI, *n* (%)	13 (24.5)	8 (28.6)	5 (20.0)	0.54
Coronary angiography, *n* (%)¹	43 (81.1)	23 (82.1)	20 (80.0)	1.00
1-vessel CAD	6 (14.0)	4 (17.4)	2 (10.0)	0.67
2-vessel CAD	5 (11.6)	4 (17.4)	1 (5.0)	0.35
3-vessel CAD	32 (74.4)	15 (65.2)	17 (85.0)	0.18
Baseline LVEF, %	33.91 ± 4.35	33.71 ± 4.64	34.12 ± 4.09	0.74
CABG-to-follow-up echo interval, days	317 [248–353]	334 [256–350]	309 [226–369]	0.87
*PET parameters*				
Mismatch index, %	3 [1 8]	2 [1 6]	5 [2 9]	0.11
Match index, %	6 [2 11]	7 [3, 16]	4 [2 9]	0.07
MMR	0.50 [0.20, 1.73]	0.32 [0.08, 0.57]	0.79 [0.48, 2.86]	**0.01**
SUV_infarct_	5.24 ± 2.25	4.86 ± 2.16	5.67 ± 2.30	0.17
SUV_non-infarct_	6.70 [5.00, 8.80]	7.25 [5.40, 8.72]	6.40 [4.20, 8.90]	0.64
SUV_blood-pool_	1.60 [1.30, 2.00]	1.60 [1.30, 2.23]	1.60 [1.30, 1.90]	0.59
NSUV_infarct_	3.15 [2.28, 4.00]	2.74 [2.00, 3.94]	3.45 [2.56, 4.16]	0.08
NSUV_non-infarct_	4.41 [2.83, 5.57]	4.32 [2.81, 5.81]	4.67 [3.25, 5.50]	0.99
SUVR	0.75 [0.60, 0.89]	0.65 [0.58, 0.81]	0.88 [0.71, 0.95]	**<0.01**

Note. Continuous variables are presented as mean ± SD or median [IQR], and categorical variables as *n* (%).

*P* values were obtained using the independent-samples t test, Wilcoxon rank-sum test, or Fisher exact test, as appropriate; bold values indicate *P* < 0.05.

¹ Coronary angiography data were available for 43 patients (ΔLVEF <10%, *n* = 23; ΔLVEF ≥10%, *n* = 20).

*Abbreviations: BMI, body mass index; CABG, coronary artery bypass grafting; PCI, percutaneous coronary intervention; CAD, coronary artery disease; IQR, interquartile range; LVEF, left ventricular ejection fraction; MI, myocardial infarction; MMR, mismatch-to-match ratio; NSUV, normalized standardized uptake value; PET, positron emission tomography; SD, standard deviation; SUV, standardized uptake value; SUVR, infarct-to-non-infarct SUV ratio.*

In separate Firth models adjusted for age, baseline LVEF, history of CAD, and diabetes, SUVR was associated with both LVEF outcomes. Each 0.1-unit increase in SUVR was associated with an OR of 1.94 (95% CI 1.28–3.25; *P* < 0.001; *q* = 0.003) for ΔLVEF ≥5% and an OR of 1.53 (95% CI 1.11–2.29; *P* = 0.004; *q* = 0.035) for ΔLVEF ≥10% (Table 3). The match index and NSUV_infarct_ were also associated with ΔLVEF ≥5% after correction; neither remained significant for ΔLVEF ≥10%.

**Table 3:** Multivariable Firth-penalized logistic regression of PET parameters and postoperative LVEF recovery.

Variables	ΔLVEF ≥5%			ΔLVEF ≥10%		
	OR (95% CI)	*P value*	BH *q* value	OR (95% CI)	*P value*	BH *q* value
Mismatch index, %	2.38 (0.78–9.38)	0.132	0.237	2.23 (0.79–7.15)	0.130	0.292
Match index, %	0.33 (0.12–0.75)	**0.007**	**0.033**	0.43 (0.17–0.96)	0.039	0.176
MMR	1.17 (0.95–1.77)	0.200	0.299	1.09 (0.96–1.34)	0.205	0.307
SUV_infarct_	1.30 (0.98–1.81)	0.065	0.146	1.19 (0.93–1.57)	0.178	0.307
SUV_non-infarct_	0.92 (0.75–1.12)	0.403	0.518	0.93 (0.77–1.12)	0.457	0.587
SUV_blood-pool_	0.91 (0.43–1.78)	0.788	0.870	0.97 (0.47–1.88)	0.921	0.921
NSUV_infarct_	1.87 (1.10–3.64)	**0.014**	**0.043**	1.40 (0.98–2.41)	0.064	0.192
NSUV_non-infarct_	1.02 (0.80–1.32)	0.870	0.870	1.01 (0.79–1.28)	0.913	0.921
SUVR	1.94 (1.28–3.25)	**<0.001**	**0.003**	1.53 (1.11–2.29)	**0.004**	**0.035**

Note. Each model included one PET parameter and was adjusted for age, baseline LVEF, history of CAD, and diabetes. ORs are reported per 10-unit increase in the mismatch or match index, both expressed as percentages of the LV, per 0.1-unit increase in SUVR, and per unit increase in the other continuous PET parameters.

Benjamini–Hochberg false-discovery-rate correction was applied across the nine PET models within each LVEF outcome.

*Abbreviations: BH, Benjamini–Hochberg; CI, confidence interval; LVEF, left ventricular ejection fraction; MMR, mismatch-to-match ratio; NSUV, normalized standardized uptake value; OR, odds ratio; SUV, standardized uptake value; SUVR, infarct-to-non-infarct SUV ratio.*

**Fig 4:**
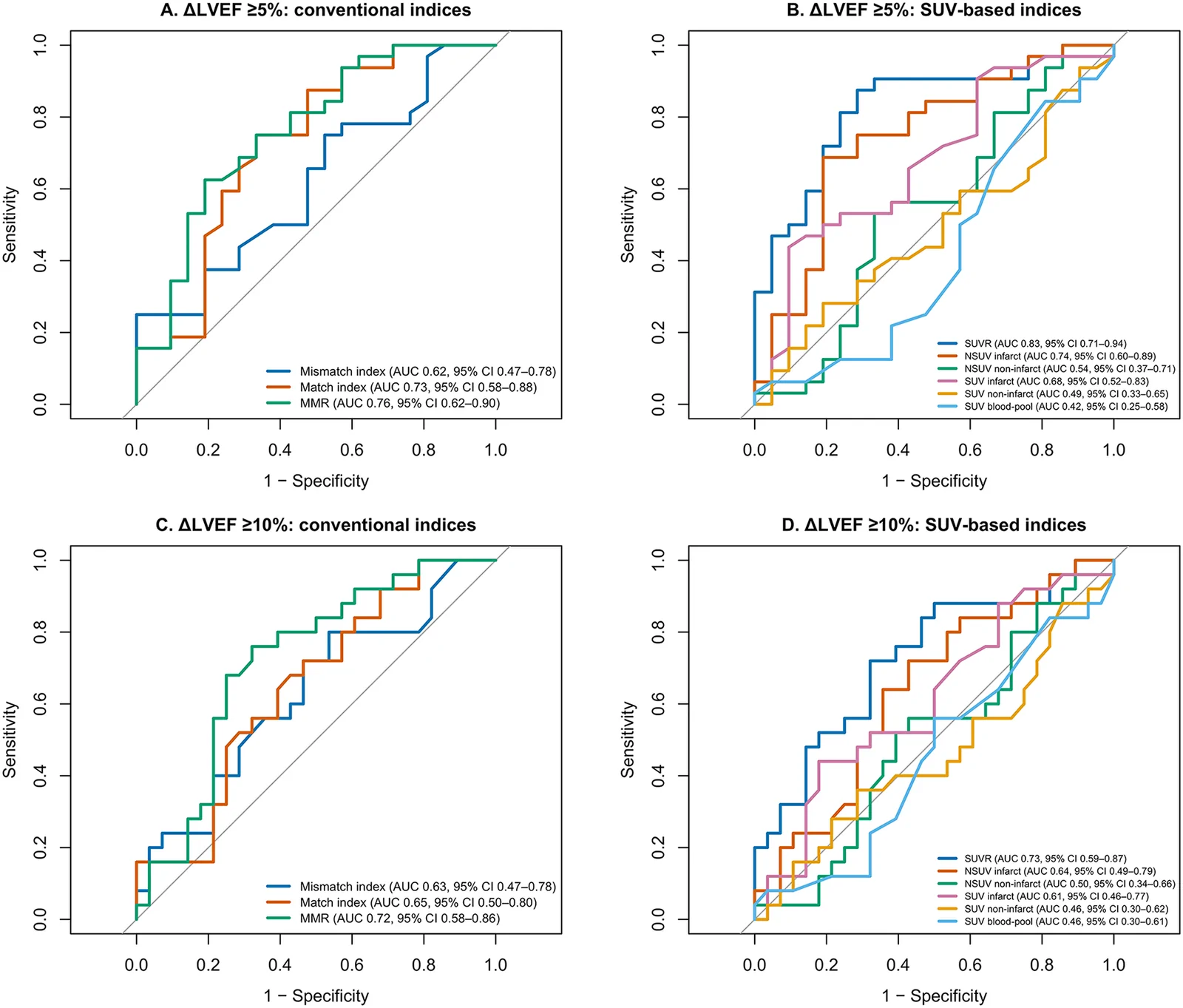
ROC curves comparing conventional perfusion–metabolism indices and SUV-based indices for postoperative LVEF recovery. **Panels A and B show ΔLVEF ≥5%, and panels C and D show ΔLVEF ≥10%; conventional indices are shown in A and C, and SUV-based indices in B and D.** Abbreviations: LVEF, left ventricular ejection fraction; MMR, mismatch-to-match ratio; NSUV, normalized standardized uptake value; ROC, receiver operating characteristic; SUV, standardized uptake value; SUVR, infarct-to-non-infarct SUV ratio.

For ΔLVEF ≥5%, SUVR had the highest AUC (0.83, 95% CI 0.71–0.94), with 88% sensitivity and 71% specificity at the exploratory cutoff of 0.66 (Fig 4; Table 4). SUVR discriminated better than the mismatch index (AUC 0.62; DeLong *P* = 0.0049; *q* = 0.0147), whereas comparisons with the match index and MMR were not significant after correction. The apparent AUC of the adjusted SUVR model was 0.84; bootstrap optimism was 0.066, yielding an optimism-corrected AUC of 0.77 (S2 Table).

**Table 4:** ROC analysis of PET parameters for LVEF recovery after CABG.

Variables	LVEF improvement ≥5%				LVEF improvement ≥10%			
	AUC (95% CI)	Cutoff	Sensitivity	Specificity	AUC (95% CI)	Cutoff	Sensitivity	Specificity
Mismatch index	0.62 (0.47–0.78) ^*^	12.85%	25%	100%	0.63 (0.47–0.78)	1.90%	80%	46%
Match index	0.73 (0.58–0.88)	6.95%	75%	67%	0.65 (0.50–0.80)	6.95%	72%	54%
MMR	0.76 (0.62–0.90)	0.60	63%	81%	0.72 (0.58–0.86)	0.45	76%	68%
SUVR	0.83 (0.71–0.94)	0.66	88%	71%	0.73 (0.59–0.87)	0.73	72%	68%
NSUV_infarct_	0.74 (0.60–0.89)	3.23	69%	81%	0.64 (0.49–0.79)	2.87	72%	57%

Note. The asterisk indicates the AUC comparison between the mismatch index and SUVR for ΔLVEF ≥5% (DeLong *P* = 0.0049; FDR-adjusted *q* = 0.0147). The corresponding comparisons of SUVR with the match index and MMR were not significant after correction. Cutoffs were derived using the Youden index and should be interpreted as exploratory.

*Abbreviations: AUC, area under the ROC curve; CABG, coronary artery bypass grafting; CI, confidence interval; LVEF, left ventricular ejection fraction; MMR, mismatch-to-match ratio; NSUV, normalized standardized uptake value; PET, positron emission tomography; SUVR, infarct-to-non-infarct SUV ratio.*

For ΔLVEF ≥10%, the AUC was 0.73 for SUVR and 0.63 for the mismatch index; the difference was not significant (DeLong *P* = 0.200; *q* = 0.383) (Fig 4; Table 4). Bootstrap validation of the adjusted SUVR model yielded an optimism-corrected AUC of 0.65, and the 2.5th–97.5th percentile cutoff range was 0.646–0.944, indicating threshold uncertainty (S2 Table). These findings for ΔLVEF ≥10% should therefore be interpreted cautiously given the limited sample size.

The SUVR estimates were materially unchanged after adjustment for the actual echocardiography interval and, among 43 patients with complete angiographic data, after additional adjustment for three-vessel CAD (S3 Table). Complete angiographic data were unavailable for the remaining 10 patients because their coronary angiography had been performed at outside hospitals and the available records were incomplete.

### Association of PET quantitative parameters with time to first MACE

**Fig 5:**
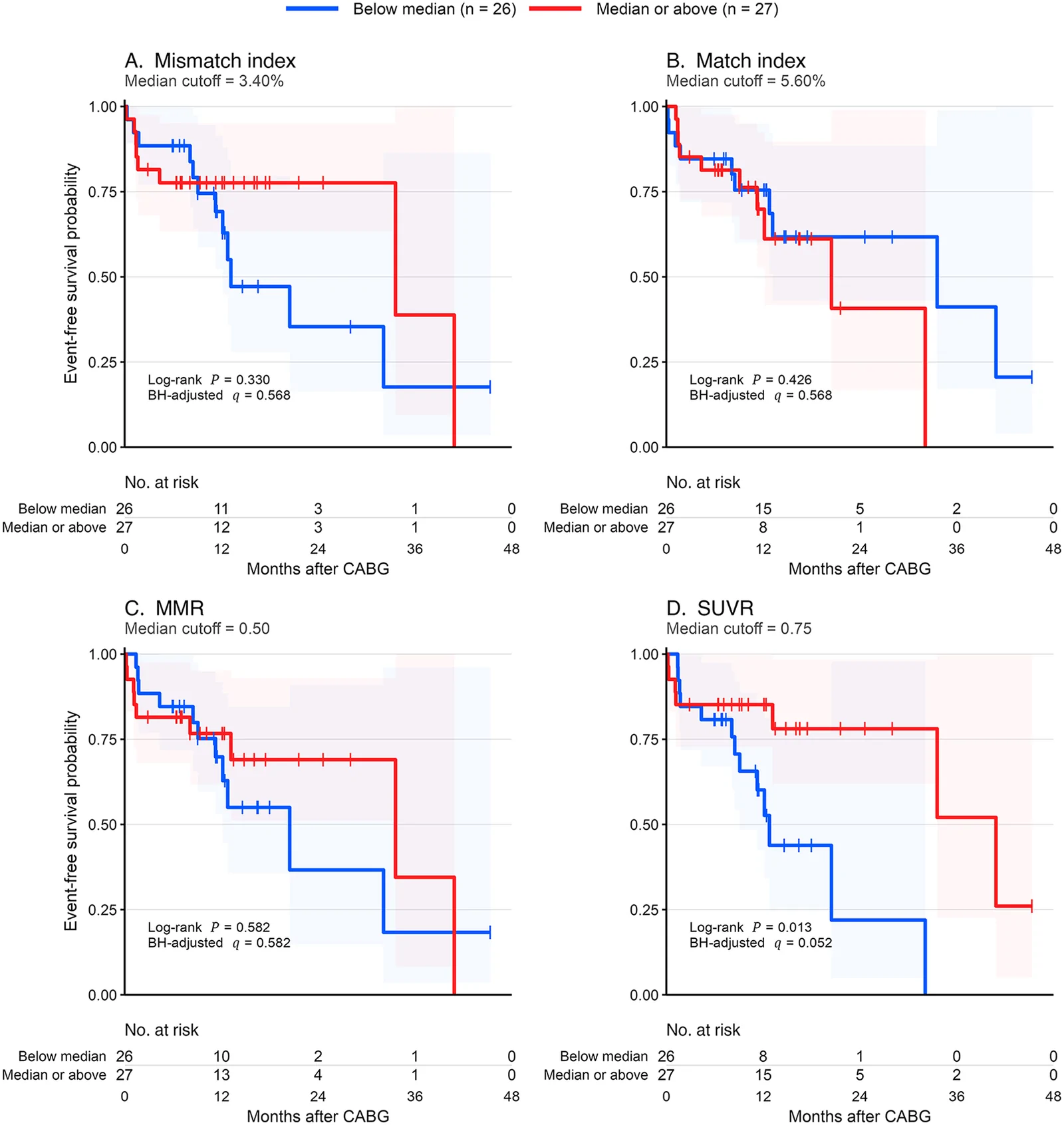
Event-free survival according to median-dichotomized PET parameters. **(A) mismatch index, (B) match index, (C) MMR, and (D) SUVR.** Shading denotes 95% confidence intervals, and P values are from log-rank tests. Abbreviations: MMR, mismatch-to-match ratio; PET, positron emission tomography; SUVR, infarct-to-non-infarct SUV ratio.

Twenty patients experienced a first MACE during a median follow-up of 11.27 months [IQR 6.51–14.85]. No PET parameter was associated with time to first MACE in Cox models adjusted for age and baseline LVEF after FDR correction (S4 Table). Continuous SUVR was not associated with MACE (hazard ratio 1.00 per 0.1-unit increase, 95% CI 0.83–1.21; *P* = 0.974; *q* = 0.974). Median-dichotomized SUVR showed a raw log-rank *P* = 0.013, but this did not meet the FDR threshold across the four Kaplan–Meier comparisons (*q* = 0.052) (Fig 5).

## Discussion

The principal finding of this study is that SUVR, defined as the ratio of [¹⁸F]FDG uptake in low-perfusion myocardium to that in non-infarct myocardium, remained associated with postoperative LVEF recovery at both ΔLVEF thresholds (≥5% and ≥10%) after prespecified clinical adjustment. For ΔLVEF ≥5%, SUVR showed greater discrimination than the conventional perfusion–metabolism mismatch index (AUC 0.83 vs 0.62), with moderate discrimination retained after bootstrap internal validation. For ΔLVEF ≥10%, however, the AUC difference between SUVR and the mismatch index was not significant, and the optimism-corrected AUC was only 0.65, indicating limited discrimination. No PET parameter was associated with time to first MACE in the adjusted Cox models. These findings support SUVR as a quantitative adjunct to conventional PET viability indices for estimating functional recovery, whereas its association with clinical outcomes remains unconfirmed.

### Traditional perfusion–metabolism indices and LVEF improvement

In the present cohort, the conventional mismatch index showed limited ability to predict postoperative LVEF improvement. Rather than indicating that myocardial viability is clinically unimportant, this finding more likely reflects a combination of cohort characteristics and methodological constraints in conventional relative uptake analysis. On the one hand, most patients in the study cohort had advanced left ventricular remodeling, resulting in a low median mismatch index (3%), which inherently limits the discriminative capacity of this semiquantitative index. On the other hand, perhaps more importantly, traditional indices relying on relative uptake of perfusion and metabolism have intrinsic methodological limitations that may compromise the sensitivity for detecting metabolically active myocardium in severely hypoperfused regions. The mismatch and match indices are derived by comparing relative uptake polar maps with a normal database [18]; as such, they primarily quantify the percentage of LV myocardium assigned to each classified category rather than the continuous spectrum of residual metabolic activity within dysfunctional myocardium.

As highlighted by Johnson and Gould [19], interpretation of [¹⁸F]FDG PET viability imaging based on relative scaling or contrast thresholds may lead to misclassification, particularly in regions with heterogeneous metabolic distribution or mixed scar–viable tissue composition. In the setting of severe hypoperfusion or extensive scarring, such contrast- and threshold-based approaches may effectively compress interregional contrast differences, further reducing sensitivity for identifying potentially recoverable myocardium.

In contrast to the mismatch index, the match index demonstrated a more consistent negative association with functional recovery in our study. Patients who experienced LVEF improvement had significantly lower match-index values, and the match index showed moderate discriminative performance on ROC analysis, suggesting that it partly reflects the burden of irreversible injury or myocardial scar. This observation is concordant with evidence from recent randomized trials and imaging studies. In REVIVED-BCIS2 and its prespecified subgroup analyses [20], scar burden was strongly and inversely associated with left ventricular functional improvement and clinical outcomes, whereas stratification based solely on the extent of hibernating myocardium did not reliably identify patients who derived greater functional or survival benefit from revascularization. Similarly, studies using late gadolinium enhancement cardiac magnetic resonance have demonstrated that the extent of preoperative scar or infarct size is a robust negative predictor of postoperative LVEF improvement and reverse remodeling [16,21]. Extensive or transmural scarring may impose mechanical constraints by increasing myocardial stiffness, disrupting contractile synchrony, and limiting favorable geometric remodeling, even when residual viable myocardium persists within or adjacent to scarred regions. However, LGE-CMR was not systematically available in this cohort; the present study therefore does not compare SUVR directly with contemporary CMR viability assessment.

In this context, MMR can be interpreted as an integrated measure of the relative balance between viable myocardium and scar burden. In our cohort, patients with LVEF improvement exhibited significantly higher MMR values, and MMR showed modest discriminative ability on ROC analysis, indicating that a higher proportion of viable myocardium relative to scar burden is associated with greater potential for functional recovery. Although MMR did not achieve statistical significance as an independent predictor in its separate multivariable model, its overall performance underscores a key concept: the functional benefit of revascularization is determined not merely by the presence of viable myocardium, but by the balance between viable tissue and irreversible scar.

### SUV-based indices and LVEF improvement

LVEF reflects global systolic function and is closely associated with hard endpoints such as mortality and hospitalization [22]. Accordingly, LVEF is endorsed as a key parameter for risk stratification and therapeutic decision-making in ESC/ACC guidelines [23]. Many PET, SPECT, and CMR studies have used absolute ΔLVEF thresholds of 5%–10% to define clinically meaningful functional improvement [24]. The STICH trial and subsequent analyses showed that LVEF improvement after CABG may not be linearly related to long-term survival benefit, even in patients with viable myocardium. LVEF was therefore used here as the functional recovery outcome rather than as a surrogate for clinical benefit.

In this study, a prespecified threshold of 50% of the maximum [^13^N]ammonia polar-map intensity defined low- and higher-perfusion regions. Prior [^13^N]ammonia work used <50% of maximal uptake to denote severely reduced perfusion [14]; here it was an operational relative-uptake boundary rather than histopathologic proof of infarction. Alternative thresholds were not evaluated. Unlike conventional relative perfusion–metabolism indices, SUV provides a body weight–normalized measure of tracer concentration. [¹⁸F]FDG uptake has been associated with functional recovery [25]. In our cohort, SUV_infarct_ showed moderate discrimination, whereas relative indices performed better, with SUVR yielding the highest AUC (0.83).

Several mechanisms may explain the stronger performance of SUV-based indices in our study. First, absolute SUV measurements are influenced by multiple biological and technical factors, including blood glucose level, uptake time, acquisition conditions, and reconstruction methods. Using blood pool activity or remote non-infarct myocardium as an internal reference may therefore partially reduce inter-scan and inter-individual variability [26]. In addition, because global myocardial [¹⁸F]FDG distribution in chronic ICM may be altered by heart failure-related metabolic remodeling, diabetes, and ongoing medical therapy, the infarct-to-non-infarct SUV ratio may provide a more stable representation of regional metabolic preservation within dysfunctional myocardium. This may explain why SUVR showed the strongest association with postoperative LVEF improvement in our cohort. Experimental pacing-induced heart failure has also demonstrated dissociation among regional glucose uptake, perfusion, and contractile dysfunction [27], supporting the biological rationale for quantitative metabolic assessment. The magnitude of the clinical association nevertheless requires confirmation in a larger cohort.

### SUV-based indices and short-term clinical outcomes

During a median follow-up of approximately 11 months, 20 patients experienced a first MACE. The raw Kaplan–Meier comparison suggested separation by median SUVR (*P* = 0.013), but it did not remain significant after FDR correction (*q* = 0.052), and continuous SUVR was not associated with MACE in the adjusted Cox model. With only 20 events, these analyses do not establish a prognostic association. This finding is consistent with the broader uncertainty regarding viability testing and clinical outcomes observed in STICH and PARR-2 [4,28]. Larger cohorts with longer follow-up are required to determine whether SUVR has prognostic value beyond its association with LVEF recovery.

### Limitations

This study has limitations inherent to its retrospective, single-center design. The modest, predominantly male cohort and inclusion of patients with complete postoperative follow-up limit generalizability and may introduce selection bias. Follow-up echocardiography timing varied within the clinically targeted window, although timing-adjusted estimates were similar. Sensitivity analyses and bootstrap internal validation strengthened model assessment but cannot replace external validation. The exploratory SUVR cutoff, absence of formal repeatability estimates, lack of systematic LGE-CMR comparison, and use of a single perfusion threshold warrant confirmation in future studies. The limited number of MACE events constrained the prognostic analysis.

## Conclusions

SUVR showed greater discrimination for postoperative ΔLVEF ≥5% than the conventional perfusion–metabolism mismatch index. This finding supports SUVR as a quantitative adjunct to conventional PET viability assessment and warrants further multicenter validation.

## Supporting information

S1 Text Detailed PET/CT acquisition, patient preparation, reconstruction, and image-analysis protocol. (DOCX)

S1 Fig Software-assisted PET segmentation and polar-map workflow. (DOCX)

S2 Fig Regions of interest and formulas for SUVR and NSUV. (DOCX)

S1 Table Unadjusted PET parameter comparisons for postoperative LVEF recovery, with Benjamini–Hochberg *q* values. (DOCX)

S2 Table Bootstrap internal validation of the adjusted SUVR models and exploratory SUVR cutoffs. (DOCX)

S3 Table Sensitivity analyses of the SUVR association after adjustment for echocardiography timing and three-vessel CAD. (DOCX)

S4 Table Adjusted Cox models for time to first MACE, with Benjamini–Hochberg *q* values. (DOCX)

S1 Data Deidentified individual-level dataset supporting all reported analyses. (XLSX)

S1 Code Reproducible R code for Firth models, FDR correction, ROC comparisons, bootstrap validation, sensitivity analyses, and survival analyses (R). (R)

S2 Code Reproducible R code for figure generation (R). (R)
