# Supplementary material for: Quantitative [13N]ammonia/[18F]FDG PET/CT predicts left ventricular functional recovery after CABG in ischemic cardiomyopathy: An exploratory study
Source: PLoS One. 2026 Sep 21;21(9):e0358657. doi: 10.1371/journal.pone.0358657 (PMC13592635; doi:10.1371/journal.pone.0358657)
Supplement: Supplementary file 1 [file pone.0358657.s001.docx]

**Supporting Information**

*Quantitative [¹³N]ammonia/[¹⁸F]FDG PET/CT Predicts Left Ventricular Functional Recovery After CABG in Ischemic Cardiomyopathy: An Exploratory Study*

**S1 Text. Detailed PET/CT acquisition, patient preparation, reconstruction, and image-analysis protocol**

**PET/CT acquisition and reconstruction**

All 53 patients underwent resting [¹³N]ammonia perfusion and [¹⁸F]FDG metabolic PET/CT on a Discovery Elite PET/CT system (NM690, GE HealthCare) equipped with time-of-flight capability and three-dimensional list-mode acquisition. Both radiotracers were produced on site using a MINItrace Qilin medical cyclotron and synthesized with a TraceLab automated synthesis system; radiochemical purity was ≥95%. The perfusion and metabolic studies were acquired consecutively on the same day within the same cardiac field of view, with [¹³N]ammonia imaging performed first.

For resting perfusion imaging, a CT localizer was used to position the heart, followed by low-dose CT (120 kV, 20 mA) for attenuation correction. [¹³N]ammonia (370–555 MBq) was administered as an intravenous bolus 10 seconds before a 10-minute dynamic PET acquisition. After a 2-minute delay, ECG-gated perfusion PET was acquired for 8 minutes with 8 frames per cardiac cycle. Images were reconstructed using an ordered-subset expectation maximization iterative algorithm and corrected for attenuation, scatter, random coincidences, and geometric efficiency.

**[¹⁸F]FDG patient preparation and acquisition**

Patients fasted for at least 6 hours; water was not restricted. Baseline blood glucose was measured, and myocardial glucose loading followed a local oral glucose–insulin protocol stratified by diabetes status. A 50% glucose solution was supplied in 20-mL ampoules containing 10 g glucose, diluted with water, and administered orally. The dose selected from the institutional schedule ranged from 5 to 40 g according to baseline glucose and diabetes status, as summarized below.

**Oral glucose-loading schedule**

| **Diabetes status** | **Baseline blood glucose (mmol/L)** | **Oral 50% glucose solution** |
| --- | --- | --- |
| No diabetes | <6.0 | 40 g |
| No diabetes | 6.0–7.0 | 30–35 g |
| No diabetes | 7.0–8.0 | 15–20 g |
| No diabetes | 8.0–9.0 | 5–10 g |
| Diabetes | Approximately 6.0 | 20 g |
| Diabetes | 7.0–8.0 | 15–20 g |
| Diabetes | 9.0–10.0 | 10–15 g |
| Diabetes | >10.0 | 5 g; insulin could be used directly when markedly elevated |

The dose ranges reflect the local preparation schedule used during the study period.

Blood glucose was remeasured approximately 60 minutes after oral glucose. Regular insulin (400 IU/10 mL stock solution) was diluted with normal saline and administered slowly by the intravenous route when indicated. The institutional schedule used doses of 0.5–4 IU according to the measured glucose concentration and diabetes status; repeat glucose testing was performed approximately 30–40 minutes after insulin administration. The protocol specified a maximum insulin dose of 4 IU, with any additional dosing considered cautiously only after repeat testing. Patients were monitored for symptoms of hypoglycemia throughout preparation.

**Post-loading insulin and tracer-injection schedule**

| **Diabetes status** | **Assessment time** | **Blood glucose (mmol/L)** | **Protocol action** |
| --- | --- | --- | --- |
| No diabetes | Approximately 60 min | 7–8 | 0.5 IU insulin; tracer injection permitted |
| No diabetes | Approximately 60 min | 8–9 | 0.5–1 IU insulin; tracer injection permitted |
| No diabetes | Approximately 60 min | 9–10 | 2 IU insulin; delay and remeasure |
| No diabetes | Approximately 60 min | ≥11 | 3–4 IU insulin; delay and remeasure |
| Diabetes | Approximately 60 min | Approximately 10 | 2 IU insulin; delay and remeasure |
| Diabetes | Approximately 60 min | 11–13 | 3 IU insulin; delay and remeasure |
| Diabetes | Approximately 60 min | ≥14 | 4 IU insulin; delay and remeasure |
| Either group | 90–100 min | 7–8, lower, or clearly falling | Inject [¹⁸F]FDG |
| Either group | 90–100 min | 8–9 | 1–2 IU insulin, then inject [¹⁸F]FDG |
| Either group | 90–100 min | Continued rise | No immediate additional insulin; wait 15–20 min and remeasure |
| Either group | Approximately 120 min | 7–8, lower, or clearly falling | Inject [¹⁸F]FDG |
| Either group | Approximately 120 min | 8–9 | 1–2 IU insulin, then inject [¹⁸F]FDG |

The protocol-defined tracer-injection criterion was generally a blood glucose concentration of 7–8 mmol/L or a clear downward trend. At 8–9 mmol/L, tracer injection was permitted after low-dose insulin. Clinical judgment and repeat glucose testing were used when concentrations remained higher or continued to rise.

[¹⁸F]FDG (111–185 MBq) was administered after the protocol-defined criterion was met. A small amount of food was permitted 15–20 minutes after tracer injection. ECG-gated metabolic PET began approximately 60 minutes after injection and used the same CT and PET acquisition settings as the perfusion study, with 8 frames per cardiac cycle and an 8-minute acquisition.

**Image processing and quantitative analysis**

Attenuation-corrected [¹³N]ammonia and [¹⁸F]FDG datasets were imported into MyoFlowQ (Beijing Lark Cloud Biomedicals, Beijing, China) and reoriented to standard short-axis slices. [¹⁸F]FDG images were converted to standardized uptake value (SUV) using image-derived activity concentration (Bq/mL), injected activity (Bq), and body weight. A valve-plane cut line and an ellipsoidal left-ventricular sampling surface were used to generate aligned perfusion and metabolic polar maps.

The prespecified low-perfusion mask comprised pixels with <50% of the maximum [¹³N]ammonia intensity; the remaining pixels formed the non-infarct reference region. This 50% boundary was an operational relative-uptake convention supported by prior [¹³N]ammonia work [14], rather than a histopathologic definition of infarction, and it was not optimized against postoperative LVEF. The mask was transferred to the [¹⁸F]FDG polar map to obtain mean SUV within the low-perfusion and remaining myocardial regions. A 10 × 10 × 20 mm³ rectangular ROI in the left atrial cavity provided the blood-pool reference.

The derived parameters were SUVR = SUV_infarct_/SUV_non-infarct_, NSUV_infarct_ = SUV_infarct_/SUV_blood-pool_, and NSUV_non-infarct_ = SUV_non-infarct_/SUV_blood-pool_. Conventional mismatch and match indices, each expressed as a percentage of LV myocardium, were obtained using the PARR-1 method [15], and the mismatch-to-match ratio (MMR) was calculated. Two experienced nuclear medicine physicians, blinded to clinical history, surgical details, and postoperative echocardiographic and clinical outcomes, jointly reviewed image registration, myocardial contours, ROI placement, and final measurements. Any discrepancies were resolved by consensus before measurements were finalized for analysis. MyoFlowQ has not undergone published head-to-head validation against QGS/QPS for this workflow.

Reference numbers correspond to the reference list in the main manuscript.
