# Supplementary material for: Quantitative [13N]ammonia/[18F]FDG PET/CT predicts left ventricular functional recovery after CABG in ischemic cardiomyopathy: An exploratory study
Source: PLoS One. 2026 Sep 21;21(9):e0358657. doi: 10.1371/journal.pone.0358657 (PMC13592635; doi:10.1371/journal.pone.0358657)
Supplement: Supplementary file 2 [file pone.0358657.s002.docx]

**S1 Fig. Software-assisted PET segmentation and polar-map workflow**

The workflow summarizes import and short-axis reorientation of attenuation-corrected perfusion and metabolic images, generation and alignment of polar maps, calculation of the conventional mismatch and match indices, transfer of the prespecified low-perfusion mask for regional SUV sampling, and final consensus review by two nuclear medicine physicians. Abbreviations: LV, left ventricle; MMR, mismatch-to-match ratio; PET, positron emission tomography; SUV, standardized uptake value.
