# Supplementary material for: Quantitative [13N]ammonia/[18F]FDG PET/CT predicts left ventricular functional recovery after CABG in ischemic cardiomyopathy: An exploratory study
Source: PLoS One. 2026 Sep 21;21(9):e0358657. doi: 10.1371/journal.pone.0358657 (PMC13592635; doi:10.1371/journal.pone.0358657)
Supplement: Supplementary file 3 [file pone.0358657.s003.docx]

**S2 Fig. Regions of interest and formulas for SUVR and NSUV**

Low-perfusion (<50% of maximum [¹³N]ammonia uptake) and remaining-myocardial regions were transferred to the [¹⁸F]FDG map, and a left atrial ROI provided the blood-pool reference. SUVR = SUV_infarct_/SUV_non-infarct_; NSUV_infarct_ = SUV_infarct_/SUV_blood-pool_; and NSUV_non-infarct_ = SUV_non-infarct_/SUV_blood-pool_. Abbreviations: LV, left ventricle; NSUV, normalized standardized uptake value; ROI, region of interest; SUV, standardized uptake value; SUVR, infarct-to-non-infarct SUV ratio.
