# Supplementary material for: Quantitative [13N]ammonia/[18F]FDG PET/CT predicts left ventricular functional recovery after CABG in ischemic cardiomyopathy: An exploratory study
Source: PLoS One. 2026 Sep 21;21(9):e0358657. doi: 10.1371/journal.pone.0358657 (PMC13592635; doi:10.1371/journal.pone.0358657)
Supplement: Supplementary file 4 [file pone.0358657.s004.docx]

**S2 Table. Bootstrap internal validation of the adjusted SUVR models and exploratory SUVR cutoffs**

| **Endpoint** | **Univariable SUVR AUC** | **Apparent adjusted model AUC** | **Mean optimism** | **Optimism-corrected AUC** | **Optimism-corrected AUC percentile range** | **Bootstrap median SUVR cutoff** | **Cutoff percentile range** |
| --- | --- | --- | --- | --- | --- | --- | --- |
| ΔLVEF ≥5% | 0.827 | 0.836 | 0.066 | 0.770 | 0.671–0.881 | 0.655 | 0.640–0.878 |
| ΔLVEF ≥10% | 0.727 | 0.737 | 0.091 | 0.646 | 0.520–0.775 | 0.733 | 0.646–0.944 |

Note. The adjusted Firth models included SUVR, age, baseline LVEF, history of CAD (prior myocardial infarction and/or prior percutaneous coronary intervention), and diabetes. Optimism correction was based on 1,000 valid bootstrap resamples per outcome. The optimism-corrected AUC and cutoff percentile ranges are the 2.5th–97.5th percentiles of the bootstrap distributions and are not external-validation confidence intervals. SUVR cutoffs were selected by the Youden index within each bootstrap sample and remain exploratory. Abbreviations: AUC, area under the receiver operating characteristic curve; CAD, coronary artery disease; LVEF, left ventricular ejection fraction; SUVR, infarct-to-non-infarct standardized uptake value ratio.
