# Supplementary material for: Quantitative [13N]ammonia/[18F]FDG PET/CT predicts left ventricular functional recovery after CABG in ischemic cardiomyopathy: An exploratory study
Source: PLoS One. 2026 Sep 21;21(9):e0358657. doi: 10.1371/journal.pone.0358657 (PMC13592635; doi:10.1371/journal.pone.0358657)
Supplement: Supplementary file 6 [file pone.0358657.s006.docx]

**S3 Table. Sensitivity analyses of the SUVR association after adjustment for echocardiography timing and three-vessel CAD**

| **Endpoint** | **Model** | ***n*** | **Events** | **OR per 0.1-unit SUVR (95% CI)** | ***P*** |
| --- | --- | --- | --- | --- | --- |
| ΔLVEF ≥5% | Main adjusted Firth model | 53 | 32 | 1.94 (1.28–3.25) | **<0.001** |
| ΔLVEF ≥5% | Main model plus echo follow-up interval | 53 | 32 | 1.92 (1.27–3.20) | **<0.001** |
| ΔLVEF ≥5% | Main model plus echo interval and three-vessel CAD | 43 | 25 | 1.89 (1.21–3.47) | **0.002** |
| ΔLVEF ≥10% | Main adjusted Firth model | 53 | 25 | 1.53 (1.11–2.29) | **0.004** |
| ΔLVEF ≥10% | Main model plus echo follow-up interval | 53 | 25 | 1.51 (1.10–2.26) | **0.005** |
| ΔLVEF ≥10% | Main model plus echo interval and three-vessel CAD | 43 | 20 | 1.67 (1.12–2.82) | **0.008** |

Note. The main adjusted Firth model included age, baseline LVEF, history of CAD (prior myocardial infarction and/or prior percutaneous coronary intervention), and diabetes. Echo-timing models additionally included the centered CABG-to-follow-up echocardiography interval. Models including three-vessel CAD used complete angiographic cases (*n* = 43). Abbreviations: CABG, coronary artery bypass grafting; CAD, coronary artery disease; CI, confidence interval; LVEF, left ventricular ejection fraction; OR, odds ratio; SUVR, infarct-to-non-infarct standardized uptake value ratio.
