# Supplementary material for: Quantitative [13N]ammonia/[18F]FDG PET/CT predicts left ventricular functional recovery after CABG in ischemic cardiomyopathy: An exploratory study
Source: PLoS One. 2026 Sep 21;21(9):e0358657. doi: 10.1371/journal.pone.0358657 (PMC13592635; doi:10.1371/journal.pone.0358657)
Supplement: Supplementary file 7 [file pone.0358657.s010.docx]

**S1 Table. Unadjusted PET parameter comparisons for postoperative LVEF recovery, with Benjamini–Hochberg *q* values**

| **PET parameter** | **Raw *P* ΔLVEF ≥5%** | **BH *q* ΔLVEF ≥5%** | **Raw *P* ΔLVEF ≥10%** | **BH *q* ΔLVEF ≥10%** |
| --- | --- | --- | --- | --- |
| Mismatch index | 0.143 | 0.215 | 0.113 | 0.203 |
| Match index | 0.006 | **0.013** | 0.065 | 0.189 |
| MMR | 0.001 | **0.007** | 0.006 | **0.026** |
| SUV_infarct_ | 0.033 | 0.059 | 0.170 | 0.255 |
| SUV_non-infarct_ | 0.884 | 0.884 | 0.643 | 0.723 |
| SUV_blood-pool_ | 0.302 | 0.389 | 0.591 | 0.723 |
| NSUV_infarct_ | 0.003 | **0.009** | 0.084 | 0.189 |
| NSUV_non-infarct_ | 0.649 | 0.731 | 0.986 | 0.986 |
| SUVR | <0.001 | **<0.001** | 0.005 | **0.026** |

Note. Raw *P* values correspond to the unadjusted PET parameter comparisons reported in Tables 1 and 2 of the main manuscript. Benjamini–Hochberg *q* values were calculated separately for the ΔLVEF ≥5% and ≥10% outcomes across nine PET parameters. All analyses included 53 patients; 32 achieved ΔLVEF ≥5% and 25 achieved ΔLVEF ≥10%. Bold *q* values indicate *q* < 0.05. Abbreviations: BH, Benjamini–Hochberg; LVEF, left ventricular ejection fraction; MMR, mismatch-to-match ratio; NSUV, normalized standardized uptake value; PET, positron emission tomography; SUV, standardized uptake value; SUVR, infarct-to-non-infarct SUV ratio.
